# TU-DAT: A Computer Vision Dataset on Road Traffic Anomalies

**DOI:** 10.3390/s25113259

**Published:** 2025-05-22

**Authors:** Pavana Pradeep Kumar, Krishna Kant

**Affiliations:** Computer and Information Sciences Department, Temple University, Philadelphia, PA 19122, USA

**Keywords:** intelligent transport systems, anomaly detection in road traffic

## Abstract

This paper introduces TU-DAT, a novel, freely downloadable computer vision dataset for analyzing traffic accidents using roadside cameras. TU-DAT addresses the lack of public datasets for training and evaluating models focused on automatic detection and prediction of road anomalies. It comprises approximately 280 real-world and simulated videos, collected from traffic CCTV footage, news reports, and high-fidelity simulations generated using BeamNG.drive. This hybrid composition captures aggressive driving behaviors—such as tailgating, weaving, and speeding—under diverse environmental conditions. It includes spatiotemporal annotations and structured metadata such as vehicle trajectories, collision types, and road conditions. These features enable robust model training for anomaly detection, spatial reasoning, and vision–language model (VLM) enhancement. TU-DAT has already been utilized in experiments demonstrating improved performance of hybrid deep learning- and logic-based reasoning frameworks, validating its practical utility for real-time traffic monitoring, autonomous vehicle safety, and driver behavior analysis. The dataset serves as a valuable resource for researchers, engineers, and policymakers aiming to develop intelligent transportation systems that proactively reduce road accidents.

**Dataset:** The TU-DAT dataset is accessible via GitHub 3.4 at the following URL: https://github.com/pavana27/TU-DAT (accessed on 18 May 2025). The dataset should be used only for research purposes and may not be used for profit either as it stands or with repackaging or modifications. The dataset is offered without any liability regarding any consequences resulting from using these data.

## 1. Introduction

### Need for Accident Datasets

The National Highway Traffic Safety Administration (NHTSA) estimates that about 29,135 people died in car crashes in the first nine months of 2024 [1]. The department-wide adoption of the safe system approach is the foundation of the NRSS’s implementation and is essential for resolving the fatality crisis on our roads. Roadside cameras that can nonintrusively monitor the traffic and their real-time analysis, followed by alerts to the driver (including desired maneuvers in cases of high risks of accidents), can make the roads safer.

Unfortunately, datasets for accident detection and prediction are limited by several factors: (i) traffic accidents are rare, making it impractical to gather sufficient data through prolonged recording at intersections, and (ii) legal and privacy restrictions complicate access to traffic camera footage. Due to the challenges in collecting real-world traffic accident videos—such as the rarity of events concerning accidents—many traffic cameras are operated by government agencies or private entities, making it challenging to obtain permissions or licenses to access the data.

Accident datasets are invaluable assets that underpin various aspects of road safety analysis, traffic management, and the advancement of intelligent transportation systems (ITSs). The increasing complexity of modern traffic systems necessitates a robust understanding of road safety dynamics. Accident datasets, which encapsulate detailed information about traffic collisions—including environmental conditions, human behavior, and vehicle performance—play a pivotal role in discerning patterns that contribute to road incidents. Accident datasets provide empirical evidence that aids researchers and policymakers in uncovering high-risk locations and common causes of accidents. By examining patterns in large-scale data, stakeholders can devise targeted interventions to increase road safety. For instance, these datasets can reveal environmental factors, such as weather conditions or road quality, that influence collision frequency. The work of [2] emphasizes that without these data, strategies to improve traffic safety would rely heavily on theoretical assumptions rather than solid evidence derived from real-world occurrences.

In recent years, the integration of machine learning techniques with accident datasets has gained significance. By training predictive models on historical accident data, researchers can identify accident-prone areas and recommend preventive strategies. Several studies explore how large-scale accident data and deep learning enhance autonomous vehicle safety. These datasets help train machine learning models to predict and mitigate hazards, improving decision-making in self-driving systems. By analyzing crash patterns, autonomous vehicles can adapt to risk factors like road conditions, weather, and driver behavior. The authors in [3] introduced the Integrated Ensemble Learning–Logit Model (IELLM) to improve accident severity prediction. This model integrates multiple machine learning techniques to assess risk levels and anticipate crashes in real time. Leveraging accident datasets, it enhances autonomous systems’ ability to navigate complex traffic scenarios by considering factors like speed, traffic density, and environmental conditions. These advancements help self-driving cars make data-driven decisions, reducing accidents and improving road safety.

Further, accident datasets are integral to fostering a culture of safety on the roads. By facilitating evidence-based decision-making, they support advancements in technology, policy formulation, and urban planning. Without comprehensive and high-quality accident datasets, safety improvements would rely primarily on theoretical models rather than empirical evidence, limiting their real-world effectiveness [4].

This paper introduces TU-DAT, a dataset annotated with spatiotemporal data, specifically designed for analyzing traffic accidents. TU-DAT is sourced from a variety of channels, including surveillance camera footage, publicly available accident videos on YouTube, and crash scenarios generated synthetically using a high-fidelity game simulation environment. By combining a diverse range of accident recordings, TU-DAT provides a comprehensive and multimodal dataset that captures both real-world and simulated traffic incidents, making it an invaluable resource for traffic safety research, accident prediction modeling, and training for autonomous vehicles.

A key feature of TU-DAT is its spatiotemporal annotation, which allows for precise tracking of accident dynamics, vehicle interactions, and environmental conditions over time. The dataset includes detailed metadata such as vehicle trajectories, collision points, impact severity levels, road types, and weather conditions. This information offers researchers critical insights into the causes and progression of accidents. The surveillance camera footage provides an objective, fixed-angle view of real-world crashes, while the YouTube-sourced videos present a variety of accident scenarios captured from dashcams and street cameras. The simulated crashes, created with a physics-accurate game engine, enable controlled scenario testing and augment real-world data with synthetic examples to help train machine learning models for accident detection and severity estimation. We also illustrate three different ways in which we have already used this dataset in our past work.

## 2. Related Work

### 2.1. Datasets for Modeling Road Accidents

Recent research in autonomous driving and smart cities has increasingly focused on traffic safety monitoring using computer vision and deep learning. Several benchmark datasets have been proposed in this domain.

The DoTA dataset [5] includes 4677 videos labeled across nine action categories with temporal, spatial, and categorical annotations. It also introduces a prediction consistency metric to mitigate the impact of noisy object detection and tracking. However, despite its scale, DoTA underscores the scarcity of real-world, extensively annotated accident datasets. The CADP dataset [6] comprises 1416 CCTV-recorded accidents under various weather and lighting conditions. It uses 7000 frames (balanced between accident and non-accident scenes) for training and proposes a hybrid detection method using DETR and Random Forest classifiers for frame-level classification. Ijjina et al. [7] compiled vehicular collisions from YouTube, compressed into 20 s clips at 30 FPS. These capture diverse conditions such as daylight, snow, and nighttime at intersections, but lack high-quality temporal annotations.

The QMUL Junction dataset [8] features 78,000 frames from a single suburban traffic intersection. It includes complex background activity (roads, sidewalks, buildings) and dense multi-agent interactions, making it useful for scene understanding. Simulated datasets have also emerged. Bortnikov et al. [9] used GTA V to generate annotated crash sequences under varied weather and traffic conditions. Free-range camera mods and AI tweaks allowed the authors to control scene perspective and driving behavior, achieving footage that closely resembles real-world data.

Dashcam-based datasets are also prominent. The Car Crash Dataset (CCD) [10] offers 1500 annotated clips of 5 s each, covering diverse environmental conditions. The extended CADP dataset [6] includes 4675 dashcam videos (1150 accident and 3525 normal), with annotations marking crash onset. The DAD dataset [11] includes 620 accident and 1130 regular dashcam videos from Taiwan, each sampled into 100 frames, with fixed accident timing. Beyond visual datasets, some studies [12,13] explore driver behavior using in-vehicle sensors, smartphones, and dashboard cameras to detect fatigue, distraction, and intoxication. These intrusive methods contrast with non-intrusive approaches like in our work [14], which leverage roadside CCTV for passive behavioral modeling.

Unlike prior datasets—often limited by dashcam viewpoints, fixed durations, or lack of spatiotemporal context—TU-DAT provides a hybrid real-simulated video collection with detailed annotations, multi-angle roadside views, and coverage of environmental and behavioral diversity. This enables robust benchmarking for both anomaly detection and logic-based spatiotemporal reasoning tasks.

### 2.2. Accident Detection and Prediction

In [15], the authors propose a single-class neural network technique using a Convolutional Auto Encoder (CAE) to extract robust spatiotemporal features for detecting abnormal events in crowded scenes. This technique is part of a broader trend in anomaly detection, where several deep learning methods have emerged as non-parametric alternatives for anomaly prediction. Comprehensive surveys of deep learning techniques in traffic flow analysis and prediction can be found in [16,17]. Additionally, Ref. [18] offers an in-depth discussion on deep learning methods for anomaly detection in surveillance videos, including an analysis of supervised and unsupervised approaches and open problems. Another challenge in deploying hybrid deep learning models that integrate spatial and temporal components is the discrepancy between training and prediction time horizons. While some studies, such as [19], show that hybrid deep learning architectures can improve performance under certain conditions, there is ongoing debate regarding the necessity and effectiveness of fine-tuning these models for real-world applications. The scaling of these models remains a significant concern for widespread deployment in traffic monitoring and anomaly detection systems.

## 3. Dataset Description

The TU-DAT dataset plays a crucial role in traffic accident analysis by enhancing spatiotemporal reasoning and anomaly prediction in road traffic. Unlike conventional neural networks, we have explored explicit logic-based reasoning, providing high accuracy and explainability in traffic monitoring. TU-DAT has proven instrumental in predicting and resolving traffic irregularities through the Compositional Framework for Anomaly Resolution (C-FAR). This framework combines deep learning-based object detection (YLLO) and logical reasoning (RTEC) to monitor real-time traffic conditions, anticipate potential collisions, and resolves inconsistencies in road behavior. By employing an event-driven approach, TU-DAT enables continuous risk assessment by analyzing vehicle movement patterns, inter-object distances, and road safety constraints. The epsilon-DDS algorithm, integrated into C-FAR, dynamically adjusts key variables to restore logical consistency and mitigate accident risks. Through these capabilities, TU-DAT not only enhances accident detection and prevention but also strengthens traffic management strategies and improves autonomous vehicle safety, making it a critical asset in intelligent transportation systems. Furthermore, TU-DAT has proven highly effective in enhancing vision–language models (VLMs) by integrating traditional computer vision techniques with logical reasoning. This improves situational awareness in traffic monitoring systems, enabling the detection of rare but critical incidents such as near-miss collisions and unauthorized lane changes. Thus, TU-DAT stands out as a comprehensive dataset for intelligent traffic monitoring, autonomous vehicle safety, and real-time accident prediction.

Additionally, this dataset can be used in driving education based on the analysis of traffic incident patterns like aggressive driving. This can provide insights for developing effective training programs that assist drivers in recognizing hazards and comprehending risky behaviors. Other related uses include focused awareness initiatives, advocating for safe driving practices, enhancing driver conduct, and decreasing accident rates.

### 3.1. Dataset Creation

#### 3.1.1. Data Collection

To create the dataset, we first created a Python 3.10-based crawler to extract accident videos from news and documentary websites. We also conducted searches on YouTube for various types of anomalies using text search queries with slight variations (e.g., “unexpected object on the road”, “pedestrian accident”, etc.). To ensure our approach is suitable for roadside edge devices, we exclusively use footage and images from traffic CCTV cameras. Additionally, we also utilized the BeamNG.drive [20] game simulator to generate road traffic video data. This approach allowed us to simulate aggressive driving behaviors, including speeding, tailgating, weaving through traffic, and running red lights. Using this method, we collected approximately 40 videos of positive examples (aggressive driving) and 25 videos of negative examples (non-aggressive driving). To ensure data reliability, we manually screened all collected videos to filter out irrelevant footage, low-quality streams, and videos lacking temporal consistency. We set a minimum resolution requirement for all videos and excluded clips with excessive compression artifacts. Additionally, we created simulation scenarios to replicate real-world behaviors under various lighting and terrain conditions.

#### 3.1.2. Data Annotation

We used the Computer Vision Annotation Tool (CVAT) [21] to annotate the video frames. The anomalous situation time is labeled at the time of the anomaly in temporal annotations. The dataset provides diverse real-world examples, capturing variations in visibility, road surface conditions, and vehicle interactions. This comprehensive coverage allows for more robust anomaly detection and predictive modeling by accounting for the impact of weather on traffic incidents. Spatiotemporal annotations consist of bounding boxes that identify vehicles and pedestrians, as well as time-stamped event labels (such as impact and evasive actions) and environmental metadata (including weather conditions and visibility). These annotations can provide frame-level supervision for training temporal models and support rule-based systems like Event Calculus in identifying causal patterns related to accidents.

**Figure 1:**
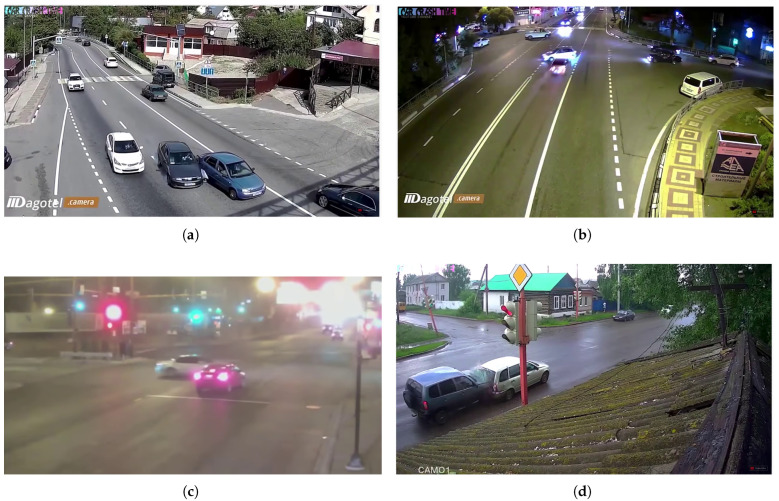

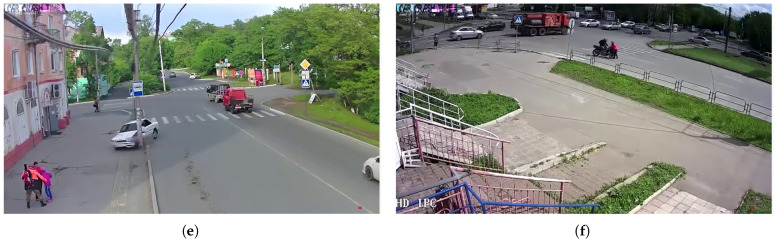
(**a**–**f**) Some frames of TU-DAT dataset of accident scenarios. (**a**) shows a side scraping between two vehicles, (**b**) shows a vehicle hitting a car attempting to cross the road, (**c**) shows a front-end accident scenario in a low-light condition, (**d**) shows a rear-end accident on a rainy day, (**e**) shows a car hitting a pole, and (**f**) shows a motorcycle hitting a pedestrian.

Figure 1 presents various crash scenarios captured under different weather conditions in our TU-DAT dataset. Figure 1a–d depict accident scenarios captured by roadside cameras under various day/night and weather conditions. Figure 1e illustrates a scenario where a car collides with a stationary object, such as an electric pole, while Figure 1f shows an accident involving a pedestrian being struck by a motorcycle. The details of our dataset are shown in Table 1.

### 3.2. Statistics of TU-DAT Dataset

**Figure 2:**
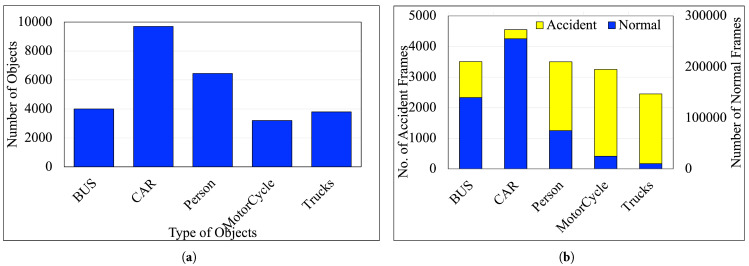
Statistics of TU-DAT dataset. (**a**) Number of objects by categories. (**b**) Number of normal/accident frames.

Figure 2a illustrates the distribution of detected objects in a dataset across six categories: Bus, Car, Person, MotorCycle, and Trucks, where the y-axis represents the total count of objects, while the x-axis denotes the object categories. The Car category comprises most objects, with approximately 140,000 detections, suggesting a substantial emphasis on traffic environments dominated by cars. This distribution signifies the dataset’s primary focus on urban or suburban traffic, making it suitable for applications like pedestrian safety analysis, accident prediction, or traffic monitoring.

Figure 2b illustrates the distribution of several accidents and regular frames in the dataset across six categories: Bus, Car, Person, MotorCycle, and Trucks, where the y-axis represents the number of frames, while the x-axis denotes the object categories. We have collected around 210 videos of road accidents varying around 24–30 FPS through these steps, with 17,255 accident frames and 505,245 regular frames.

Table 2 provides a comparative summary of the TU-DAT dataset against several prominent existing datasets, including DAD, CADP, and AI-City. This comparison includes key metrics such as the total number of videos, the average number of frames per video, camera resolution, view depth, coverage of weather variations, and the presence of annotations for foreground and background (FG/BG) activities. One of the standout features of TU-DAT is its detailed representation of both foreground and background activities. Foreground activities refer to the main events of interest—such as collisions, pedestrian impacts, tailgating, or sudden lane changes—that typically involve the primary agents in a traffic incident. In contrast, background activities encompass contextual elements, including pedestrian movements on sidewalks, vehicles in adjacent lanes, and environmental cues (e.g., traffic signals, weather conditions) that, while not directly involved in the incident, play a significant role in understanding the situation and enhancing predictive modeling.

As summarized in Table 2, existing traffic anomaly datasets exhibit several limitations, and those limitations constrain their applicability in a real-world deployment with generalizable model training. Due to offering dashcam clips of 100 frames with little spatiotemporal context, the moderately sized DAD dataset lacks environmental diversity, depth of view, and high-resolution footage. CADP includes multi-view CCTV footage; the footage has higher resolution with variable weather conditions. However, CADP does not annotate background activities, which are critical for reasoning about context-aware anomalies. AI-City provides traffic surveillance clips that last a long time and annotates them in detail; however, it does not vary the environment by omitting adverse conditions like rain, fog, or nighttime scenes. It also does not comprehensively represent background interactions. TU-DAT, in contrast, fills these critical gaps by offering rich foreground–background activity annotations with high-resolution, multi-angle footage, and diverse weather conditions. With this coverage, models tasked with spatiotemporal reasoning or behavioral analysis will have more effective training. Anomaly detection with this coverage should also improve under varied real-world scenarios.

### 3.3. BeamNG.drive Simulator

Accident simulations are essential for interpreting vehicle collisions, enhancing road safety, and facilitating research in automotive engineering and forensic accident analysis. BeamNG.drive, featuring a sophisticated soft-body physics engine, is an optimal platform for producing realistic accident videos. These videos serve multiple purposes, including driver education, insurance fraud detection, law enforcement investigations, and vehicle safety research [22]. Manufacturers and safety organizations, including the National Highway Traffic Safety Administration (NHTSA) and the European New Car Assessment Programme (Euro NCAP), depend on physical crash tests to assess vehicle safety. However, actual crash tests are costly and labor-intensive [23]. BeamNG.drive provides an economical method for virtually pre-evaluating vehicle designs before crash testing. The capacity to produce authentic accident videos with BeamNG.drive markedly improves accident analysis, safety research, driver education, and insurance fraud detection. Experts in various sectors can enhance vehicle design, road safety, and forensic accident analyses using sophisticated physics-based simulations, thereby decreasing traffic-related fatalities and injuries.

#### BeamNG.drive: Features, Physics, and Applications

**Figure 3:**
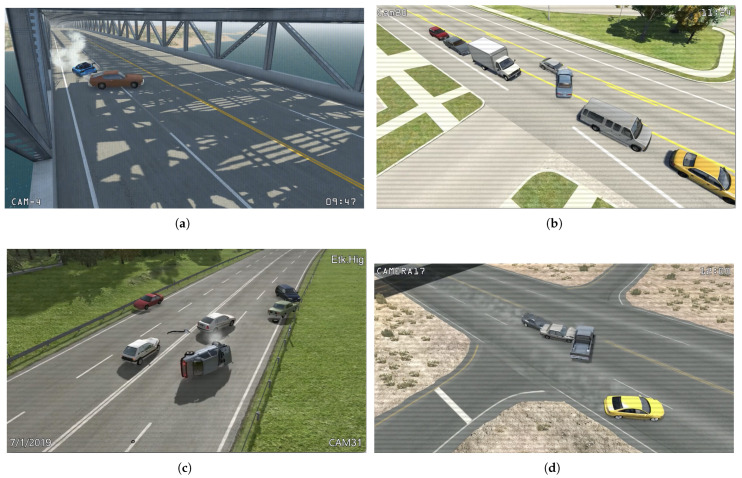
(**a**–**d**) BeamNG.drive video frames from TU-DAT dataset of accident scenarios.

BeamNG.drive is a sophisticated vehicle simulation game recognized for its realistic soft-body physics and intricate vehicle dynamics. Created by BeamNG GmbH, this simulator offers an open-world environment where players can drive, crash, and customize a variety of vehicles across different scenarios. Unlike conventional racing or driving games, BeamNG.drive prioritizes realism, making it a favored choice among automotive enthusiasts, game modders, and researchers exploring vehicle dynamics and crash scenarios. Figure 3a–d show some of the frames from videos generated using the BeamNG.drive simulator from the TU-DAT dataset. Its key features include the following:

- Soft-Body Physics Model: BeamNG.drive’s most defining feature is its unique soft-body physics engine, which determines how vehicles behave in a virtual environment. Unlike traditional rigid-body physics in most racing games, this technology allows a vehicle’s structure to deform accurately upon impact. Each part of the vehicle, from crumple zones to body panels, reacts independently and dynamically in real time to collisions. As a result, players experience a highly immersive driving simulation where vehicle physics closely mirrors real-life dynamics.
- Realistic Material Properties: At the core of BeamNG.drive’s realism is its node–beam structure, which forms the foundation of every vehicle model. Nodes represent individual physical points, while beams simulate the connections between these nodes, enabling detailed mechanical responses to external forces. This sophisticated system models materials such as steel, aluminum, plastic, and rubber, each exhibiting unique properties that influence vehicle behavior under stress. Whether it is the flex of a plastic bumper in a minor collision or the resilience of a steel frame in a high-speed crash, these material properties contribute significantly to the game’s authenticity.
- Deformation Mechanics: Deformation mechanics in BeamNG.drive enhance the impact of crashes and accidents very realistically. When a vehicle crashes, the soft-body physics engine calculates stress and strain on each component, resulting in lifelike damage representation. For instance, a high-speed collision may cause metal panels to crumple significantly, glass windows to shatter, or wheels to bend at unnatural angles. This intricate simulation also accounts for crucial factors such as impact angle, vehicle speed at the moment of collision, and the material strength of the affected components, creating a highly realistic driving experience.
- Suspension and Tire Dynamics: Beyond vehicle deformation, BeamNG.drive incorporates highly accurate suspension and tire physics, further enhancing realism. The suspension system mimics real-world behavior, effectively simulating weight transfer, body roll, and compression in actual vehicles. This attention to detail affects vehicle handling and influences how cars respond to different terrains and driving conditions. Tires interact with various surfaces—such as asphalt, gravel, and mud—realistically affecting grip levels, skidding behavior, and rolling resistance, ultimately making vehicle control challenging and immersive.
- Crash Testing Scenarios: BeamNG.drive can also be used as a tool for conducting controlled crash tests, allowing players to experiment with various vehicles in different environments. Users can simulate common crash scenarios, including head-on collisions, side impacts, rollovers, and rear-end crashes. The platform also supports AI-controlled vehicles, enabling multi-vehicle collision simulations replicating complex crash dynamics. This feature has drawn interest from engineers and researchers, who often utilize BeamNG.drive as a cost-effective way to visualize crash physics and validate safety measures before conducting real-world testing.
- Influence of Environment on Vehicle Damage: The environment plays a crucial role in BeamNG.drive’s approach to crash physics, considering multiple external factors that influence collision outcomes. For example, terrain type, surrounding obstacles, and weather conditions all impact how a vehicle behaves during a crash. A car colliding with a tree will sustain a distinctly different deformation pattern than a concrete wall impact. Additionally, off-road terrains introduce vehicle wear and tear, challenging players to navigate conditions that test their driving skills and vehicle endurance. By combining these elements, BeamNG.drive can deliver an engaging gaming experience and a rich platform for exploring vehicle physics, making it a unique offering in the world of vehicular simulation.

## 4. Methods

### 4.1. Spatiotemporal Reasoning

Before discussing the applications, we briefly talk about the spatiotemporal reasoning that we have used extensively in all these applications instead of the routine method of training a neural net. The explicit logic reasoning used in these applications has many advantages including high accuracy, explainability, and highly efficient detection appropriate for real-time monitoring of the traffic.

The spatiotemporal reasoning works by expressing all relevant events, rules of operation, rules of inference, relationships, and constraints in form of logic assertions and then using deductions to arrive at the conclusions. In particular, anomalies to be detected such as a vehicle following too closely, speeding near a stop sign, or approaching pedestrians unsafely are represented as logic assertions. We then check if any of these assertions are satisfied based on the observed behavior from the video frames or “groundings” (e.g., a car behind another one with distance decreasing) and the rules/constraints for drawing conclusions.

Basic logic reasoning can be modeled as the Boolean satisfiability problem, leveraging SMT (Satisfiability Modulo Theory) tools to expand the scope of reasoning to various other relevant “theories” such as arithmetic, motion, etc. [24]. For temporal reasoning, we employ Event Calculus (EC), which defines Events (actions at specific times) and Fluents (states affected by events). EC provides a formal framework to represent and reason about dynamic systems where the state of the world changes over time due to the occurrence of events; events cause changes in fluents, which are properties that can be true or false at different time points. This structure enables EC to accurately capture cause-and-effect relationships and temporal dependencies, which are essential for understanding and managing dynamic systems.

Event Calculus for Run-Time Reasoning (RTEC) [25] is an open-source Prolog implementation for efficient real-time event recognition, which utilizes Linear Time Logic (LTL) with integer time points to incorporate real-time aspects. RTEC extends traditional EC by supporting complex event processing, incremental reasoning, and temporal aggregation, making it well suited for dynamic environments such as real-time traffic monitoring and anomaly detection. With incremental reasoning, it allows for continuous updates as new events occur and optimizes performance through caching and indexing strategies. RTEC also provides mechanisms for event pattern recognition, temporal aggregation, and reasoning over sliding time windows, enabling it to detect temporal trends and persistent conditions critical for proactive traffic management. This allows timely detection of essential traffic conditions and enhances decision-making in dynamic environments.

### 4.2. Predicting Anomalies in Road Traffic

The primary application of the TU-DAT dataset is the prediction and resolution of anomalies in road traffic monitored through roadside camera networks. We propose a Compositional Framework for Anomaly Resolution (C-FAR) in intelligent transportation systems [26]. C-FAR focuses on predicting potential anomalies rather than detecting specific activities, accounting for uncertainty in future actions and inaccuracies in estimates like speed or distance. We handle these using weighted assertions and model the problem with Weighted Partial Maxsat (WPM) optimization. C-FAR consists of two stages, as explained in the following sections.

#### 4.2.1. Stage 1

In C-FAR, Stage 1 is a CNN-based lightweight object detection framework named YLLO that ustilizes redundancy in video to identify only the essential frames. YLLO is a three-stage process that begins with a scene change detection algorithm and progresses to object detection. Its original implementation used YOLOv6 for object detection, but it can use more recent versions of YOLO or any single-shot detector. The Simple Online and Real-time Tracking (SORT) algorithm assigns a tracker to each detected object or multiple objects. YLLO decouples classification and regression tasks to eliminate redundant objects between the frames. Additionally, before sending frames for object detection, for scene change detection, it generates Color Difference Histograms (CDHs) for edge orientations, where edge orientations are determined using the Laplacian–Gaussian edge detection framework.

#### 4.2.2. Stage 2

In Stage 2 of the C-FAR framework, we integrate an Event Calculus engine, RTEC, which processes EC predicates representing time-stamped primitive activities detected in individual video frames. These predicates encode object attributes such as bounding box coordinates, orientation, and movement direction. Each event is time-stamped, with tracked objects’ positions recorded as X and Y pixel coordinates at each time step. When dynamic dependencies are significant, developing a system capable of representing dynamic relationships is necessary. This happens in modeling accidents where the traffic conditions evolve dynamically such as a braking maneuver executed in response to danger. We define derived events as occurrences resulting from changes in state or interactions between entities. The framework detects and predicts anomalies in real time by analyzing object trajectories and spatial relationships. For instance, potential collisions are forecasted by computing inter-object distances and comparing them against predefined thresholds.

#### 4.2.3. Resolving Anomalies

Following anomaly prediction, the ϵ-DDS algorithm adjusts key variables to resolve inconsistencies. It identifies unsatisfiable constraints and modifies selected variables within a predefined threshold to restore logical consistency. This optimization process ensures that critical (or “hard”) constraints are always satisfied while the weak (or “soft”) constraints may or may not be satisfied. Each soft constraint carries a weight, and the objective is to simply maximize the sum of the weights of all satisfied soft constraints. The algorithm balances minimizing the overall perturbation and ensuring feasibility, with different penalty functions (e.g., absolute or squared differences) influencing how adjustments are distributed across variables. We develop a modified ϵ-DDS algorithm called situation-based ϵ-DDS, which compares and selects optimal solutions based on both objective function values and constraint violations. If two solutions are feasible or have similar constraint violations, the one with a better objective value is preferred. Otherwise, solutions are ranked by the degree of constraint violation, ensuring minimal disruption while effectively resolving anomalies.

### 4.3. Effectiveness of Synthetic-Real Data Fusion

The experimental results presented in this study highlight the effectiveness of combining real-world and synthetic video data for training robust anomaly detection models. Specifically, models trained on a hybrid dataset—comprising both real accident footage and simulated videos generated using BeamNG.drive—consistently outperformed those trained on either data source alone. This fusion of modalities not only improved the prediction accuracy but also enhanced the model’s ability to generalize to previously unseen accident scenarios. Notably, the model achieved a 6.8% increase in generalization performance when tested on unseen accident types, compared to a model trained solely on real-world data. These findings underscore the practical value of synthetic data augmentation in enhancing model robustness, especially in safety-critical domains where real accident data are limited or imbalanced.

### 4.4. Enhancing VLMs for Situational Awareness

We have tested our TU-DAT in our recent work [27] where we propose a novel consistency-driven fine-tuning framework for vision–language models (VLMs) [28] that integrates traditional computer vision (TCV) techniques for detailed visual recognition with explicit logical reasoning to enhance model performance. The proposed approach significantly reduces the dependency on large labeled datasets during fine-tuning by employing an intelligent input selection mechanism, resulting in substantially higher accuracy than approaches that use random or uninformed input selection.

#### 4.4.1. Fine-Tuning VLMs

Fine-tuning vision–language models (VLMs) is crucial for customizing pre-trained models to particular tasks, enhancing their precision, and honing their comprehension of multimodal data. These models, which combine textual and visual inputs, are increasingly employed in applications such as image captioning, visual question answering, and autonomous navigation [29]. Pre-trained models such as CLIP [29], Minigpt4-Video [30], and Video-LLama2 [31] are constructed using varied image–text datasets; however, they frequently lack the requisite domain-specific knowledge for specialized tasks like medical image analysis or satellite imagery interpretation [32]. Fine-tuning facilitates the adaptation of these models to specific contexts by modifying their weights through focused training on selected datasets. This process improves their capacity to identify domain-specific objects, comprehend contextual relationships, and produce more precise textual descriptions from images. These models may generate generic or erroneous outputs without fine-tuning, constraining their applicability in critical domains such as healthcare diagnostics or autonomous driving [33].

A significant limitation of fine-tuning vision–language models is the requirement for a good amount of labeled data. In contrast to natural language processing models that predominantly depend on textual corpora, vision–language models necessitate extensive, varied, and meticulously annotated image–text datasets, which can be costly and labor-intensive to assemble [29]. In specialized domains like medical imaging, we may need a large number of images and expert-annotated descriptions, necessitating domain expertise [32]. The deficiency of high-quality, annotated data in specialized domains can impede the efficacy of fine-tuning, complicating the attainment of substantial enhancements over the base model. Moreover, biased or inadequate training data may result in overfitting, wherein the model excels on the training dataset but falters in real-world generalization.

Another challenge of fine-tuning vision–language models is the computational expense. Training large-scale multimodal models necessitates high-end GPUs or TPUs, frequently rendering the process unattainable for smaller research teams or organizations with constrained resources [33]. In contrast to transfer learning in exclusively text-based models, where parameters can be efficiently fine-tuned, vision–language models (VLMs) necessitate intricate interactions between visual and linguistic elements, demanding more comprehensive optimization [29]. Despite these challenges, fine-tuning is crucial for enhancing the accuracy and applicability of vision–language models to real-world issues. Researchers persist in investigating techniques such as prompt tuning, adapter-based fine-tuning, and data-efficient learning strategies to reduce reliance on extensive labeled datasets. In the next section, we discuss an intelligent fine-tuning mechanism designed and tested using TU-DAT to address some of the challenges mentioned.

#### 4.4.2. TU-DAT in Automated Situational Understanding

Automated situational understanding is essential for video-based monitoring of cyber–physical systems and anomalous situations, such as safety issues, security breaches, policy violations, or unusual events. For instance, in traffic monitoring, key activities include identifying accidents, near-accidents, and vehicle-related criminal activities. We denote these high-level events or activities, termed main activities by the set $A^{m}$. These can be easily recognized by VLMs because of their vast pretraining base. However, finer details, such as object poses, movements, and relative locations, are crucial and are better extracted using traditional computer vision (TCV) techniques. By integrating VLMs for context and TCV for detailed analysis, supported by efficient, logical reasoning—both high-level activities and low-level details in $A^{m}$ can be effectively captured.

Two VLMs, a main VLM (VLM^*m*^) and an auxiliary VLM (VLM^*a*^), are essential for effective situational awareness. While VLM^*m*^ provides high-level activity descriptions for $A^{m}$, VLM^*a*^ complements VLM^*m*^ by identifying an auxiliary activity set ($A^{a}$) for comparison. TCV extracts finer details like object poses, movements, and relative locations which can be characterized by proxy activities ($A^{p}$) that approximate $A^{m}$ for efficient data selection. Inconsistencies between VLM^*m*^, VLM^*a*^, and TCV outputs highlight areas needing fine-tuning. This targeted approach reduces resource demands, eliminates the need for labeled data during evaluation, and enables ongoing consistency checks during inference, ensuring accurate recognition of rare but critical activities in $A^{m}$. Logical reasoning integrates these components efficiently. By analyzing inconsistencies between the outputs of VLM^*m*^, VLM^*a*^, and proxy activity recognition, we can precisely identify areas where fine-tuning is required, ensuring more targeted and efficient improvements.

The fine-tuning loop begins by evaluating a batch of inputs from the evaluation dataset (ED) of videos, called the eval-batch. This batch is processed through VLM^*m*^, VLM^*a*^, and TCV inferencing to generate outputs. These outputs are used to ground relevant assertions in the logic representation of detected classes and proxy activities, leveraging a prebuilt Logic rules database for consistency checks.

Following the grounding, SMT checks the consistency between VLM outputs and grounded assertions. If no inconsistency is found, the eval-batch is removed from ED. If inconsistencies arise, the SMT framework identifies offending assertions, pinpointing VLM classes needing further fine-tuning. The next step is selecting a batch from fine-tuning dataset (FTD) of videos, called a FT-batch, processing it, and removing it from FTD. Termination occurs if eval-batches or fine-tuning batches are exhausted, the fine-tuning time limit is exceeded, or the consistency measure stabilizes. Initially, fine-tuning uses randomly selected labeled inputs. For video-based VLMs, longer videos are segmented to focus on single-class interactions. Composite classes may also be defined for overlapping activities when necessary.

## 5. Technical Validation

### 5.1. Results of Predicting Anomalies in Road Traffic

We evaluate our proposed model by assessing its ability to anticipate future accidents or anomalies. The method computes anomaly confidence at each time slot using metrics like object distance or orientation, defined in terms of fluents or events. If the confidence at time slot *t* exceeds a threshold δ, the C-FAR framework predicts that an anomaly will occur. A correct prediction for an accident video is a True Positive (TP), where the model anticipates the accident $t^{\prime }-t$ time slots before it happens at $t^{\prime }$. For non-accident videos, such predictions are False Positives (FPs). The model predicts no anomaly if the confidence remains below δ across time slots. This results in a False Negative (FN) if the video contains an accident or a True Negative (TN) if it does not.

#### Comparison with State-of-the-Art Methods

**Figure 4:**
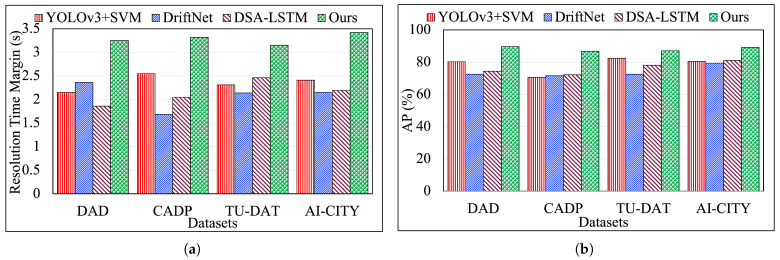
(**a**,**b**): Performance results showing RTM and AP values for all datasets from our previous work [26].

We compare our proposed model to three other existing accident detection and prediction models proposed in [11,34,35], and the performance results are shown in Figure 4a,b. In [34], the authors proposed a three-stage framework for automatic accident detection in videos. The first stage employs a car detection algorithm based on the YOLOv3 deep convolutional neural network; the second stage is a tracking algorithm based on the discriminative correlation filter method, and the final stage employs the Violent Flows (ViF) descriptor to highlight the magnitude changes in the motion vectors that are computed using an optical flow algorithm to detect car crashes. Ref. [35] is a framework for detecting anomalies, a 3D neural network architecture based on the EfficientNet 2D classifier for detecting aggressive driving, specifically car drifting. The model proposed in [11] is a Dynamic Spatial Attention (DSA)-based model that uses an RNN along with Long Short-Term Memory (LSTM) cells to model the long-term dependencies of all cues to anticipate accidents in dashcam videos.

We calculate the Average Precision (AP) from precision and recall pairs to evaluate the accuracy of our C-FAR framework. In Figure 4a, the x-axis represents datasets, and the y-axis represents the Resolution Time Margin (RTM), which is the time required to anticipate each type of anomaly in seconds, showing that C-FAR performs best on the DAD dataset with optimal AP and RTM. As seen in Figure 4a, our model anticipates accidents 3.42 s earlier with an AP of 89.27%, outperforming three other methods. The YOLOv3 + SVM method struggles with parameter setting and orientation issues, while the DSA + LSTM model is resource-intensive and inefficient. Comparing C-FAR with DriftNet, we find that C-FAR captures a broader range of driving behaviors (e.g., speeding, weaving) and achieves 95% accuracy, surpassing DriftNet’s 92.5%. Generally, C-FAR performs significantly better than all other models and on all the datasets.

### 5.2. Results on Enhancing VLMs for Situational Awareness

The proposed fine-tuning framework, henceforth called *directed finetuning* is evaluated using the TU-DAT dataset. We compare it against the *undirected fineturning* where the videos are chosen randomly for further fine-tuning. Consistency is quantified using the Consistency Improvement Factor (CIF), defined as $\left(n_{b}-n_{e}\right)/n_{b}$, where $n_{b}$ and $n_{e}$ are the inconsistencies recorded before and after fine-tuning, respectively. Directed and undirected fine-tuning methods are compared based on CIF over an equivalent number of iterations for fairness. Table 3 shows the achieved CIF for the TU-DAT dataset using X-CLIP, Video-MAE, and MiniGPT4 (image-based) and MiniGPT4-Video, Video-Llama, and VideoMamba, respectively.

#### Cross-Dataset Generalization

To evaluate the generalization capability of VLM models trained on TU-DAT, we conducted cross-dataset evaluations using three publicly available datasets: CADP, DAD, and AI-City. These datasets vary in video perspectives, environmental conditions, and annotation styles, offering a comprehensive benchmark for generalization. VLM models trained exclusively on TU-DAT were directly evaluated on each target dataset without additional fine-tuning. Table 4 presents the performance. The VLM achieves strong generalization across all benchmarks, with the highest AP on CADP 87.4% and robust performance on DAD and AI-City. These results demonstrate TU-DAT’s effectiveness as a training dataset for developing transferable models capable of handling diverse real-world traffic scenarios. The cross-dataset experiments validate the practical utility of TU-DAT beyond its native distribution. The strong performance of TU-DAT-trained VLMs on CADP, DAD, and AI-City—datasets with different camera perspectives and scene complexities—demonstrates the dataset’s effectiveness for training generalizable and transferable models. These results affirm TU-DAT’s suitability not only for anomaly detection and situational understanding but also for real-world applications in intelligent traffic monitoring and autonomous systems.

## 6. Conclusions and Future Work

In this paper, we present TU-DAT, a novel and publicly available dataset designed for analyzing road traffic accidents using computer vision techniques. TU-DAT fills a crucial gap in accessible, annotated, and diverse accident video datasets captured from roadside surveillance perspectives. By gathering real-world accident footage from news and documentary sources and augmenting it with simulated videos that model aggressive driving behavior, TU-DAT provides a rich and versatile resource for researchers. The dataset features spatiotemporal annotations, extensive modality coverage, and structured metadata, which support detailed analysis of vehicle interactions, crash dynamics, and environmental contexts. Our initial experiments with TU-DAT demonstrate the benefits of combining deep learning with logic-based spatiotemporal reasoning frameworks, yielding improvements in both accuracy and computational efficiency compared to conventional models.

We believe that TU-DAT will facilitate significant advancements in intelligent transportation systems, particularly in areas such as accident detection, predictive safety analytics, and the training of autonomous vehicle systems. By making this dataset openly available to the research community, we aim to foster innovation and collaboration toward the shared objective of improving road safety and reducing traffic-related fatalities through data-driven approaches. Future work can build upon TU-DAT by exploring several promising directions to enhance its utility for intelligent transportation research. One area of focus could be the integration of multimodal sensor data, such as LiDAR, radar, or vehicle telemetry, to provide a richer contextual understanding of accident scenarios. Furthermore, the dataset presents an opportunity to develop real-time accident prediction models that can be deployed on edge devices embedded in roadside infrastructure. Some practical challenges include legal constraints in deploying roadside cameras for real-time analysis, variability in environmental conditions affecting model robustness, and latency considerations for edge deployment. Future work should explore lightweight inference architectures and federated learning frameworks to mitigate these issues.

## Figures and Tables

**Table 1:** Statistics of TU-DAT dataset.

Conditions	No. of Frames	Accident Types	#Frames
Daylight	9796	Weaving through traffic	2417
Night/low light	1487	X-section accidents	6566
Foggy	445	Tailgating/driving maneuvers	1452/305
Rainy/snowy	128/274	Highway/rear-end accidents	1254/1215
Camera too far	211	Pedestrian accidents	447

**Table 2:** Comparison of TU-DAT dataset with other existing datasets.

Dataset	Total No. of Videos	Frames/Video	Camera Resolution	View Depth	Weather Conditions	FG/BG Activity
DAD	1730	100	No	No	No	Yes/No
CADP	1416	366	Yes	Yes	Yes	Yes/No
TU-DAT	280	960	Yes	Yes	Yes	Yes/Yes
AI-City	250	2400	Yes	Yes	No	Yes/No

**Table 3:** CIF results on TU-DAT dataset.

VLM Models	Undirected		Directed	
	VLM^m^	VLM^a^	VLM^m^	VLM^a^
X-CLIP	54.5	55.15	74.25	73.65
VideoMAE	52.04	52.41	72.65	73.25
MiniGPT4	59.78	60.41	75.51	74.35
MiniGPT4-Video	71.45	71.8	86.35	85.125
Video-Llama	72.16	72.41	86.85	87.32
VideoMamba	61.95	61.41	80.85	80.4

**Table 4:** Cross-dataset evaluation: VLM trained on TU-DAT and tested on external datasets.

Test Dataset	Model Architecture	Average Precision (AP)	F1 Score
CADP	VLM (TU-DAT-trained)	87.4%	0.843
DAD	VLM (TU-DAT-trained)	83.1%	0.812
AI-City	VLM (TU-DAT-trained)	81.8%	0.794

## Data Availability

The data described in this paper can be used for any non-commercial purpose provided that this article is clearly cited.
